# Correction: Transposable Elements Contribute to Activation of Maize Genes in Response to Abiotic Stress

**DOI:** 10.1371/journal.pgen.1005566

**Published:** 2015-10-09

**Authors:** Irina Makarevitch, Amanda J. Waters, Patrick T. West, Michelle Stitzer, Candice N. Hirsch, Jeffrey Ross-Ibarra, Nathan M. Springer

The SRA accession numbers in S1 Table are incorrect. Please view the correct S1 Table below.

## Supporting Information

S1 Table Sequencing depth for the samples used in this study. (XLSX)
