# Supplementary material for: Transposable Elements Contribute to Activation of Maize Genes in Response to Abiotic Stress
Source: PLoS Genet. 2015 Jan 8;11(1):e1004915. doi: 10.1371/journal.pgen.1004915 (PMC4287451; doi:10.1371/journal.pgen.1004915)
Supplement: S1 Fig — Most of the genes differentially expressed in response to stress have moderate expression levels in control conditions. (A) Distribution of genes up-regulated in response to stress is shown relative to their expression level (in RPKM) under control conditions. (B) Distribution of genes down-regulated in response to stress is shown relative to their expression level (in RPKM) under control conditions. (C) Distribution of genes down-regulated in response to stress is shown relative to their expression level (in RPKM) under stress conditions. (PDF) [file pgen.1004915.s001.pdf]

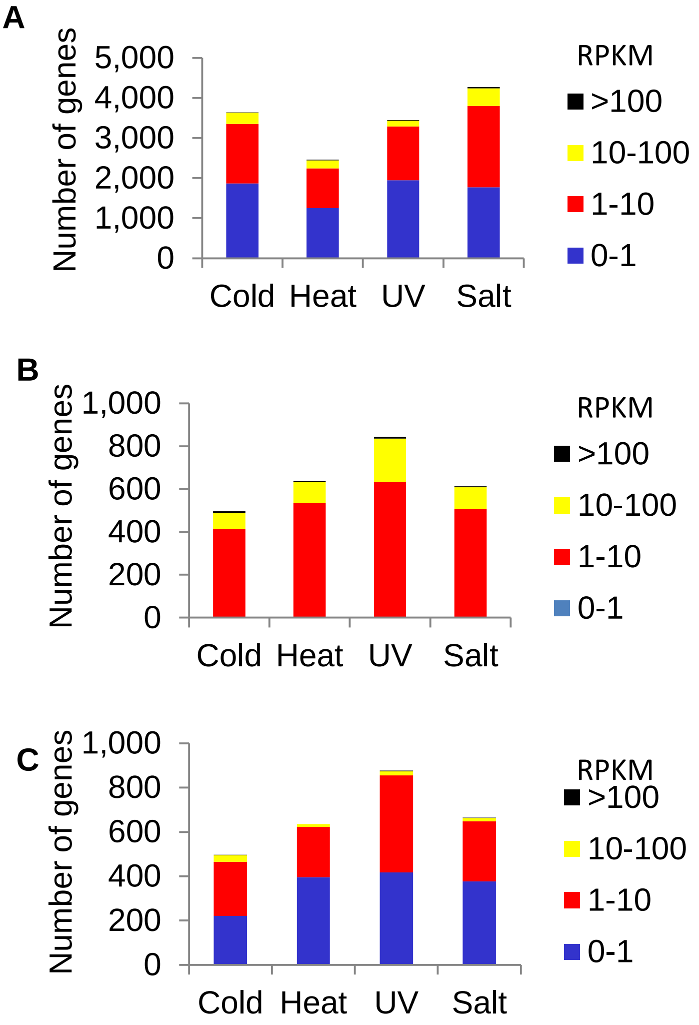

Figure S1. Most of the genes differentially expressed in response to stress have moderate expression levels in control conditions. **(A)** Distribution of genes up-regulated in response to stress is shown relative to their expression level (in RPKM) under control conditions. **(B)** Distribution of genes down-regulated in response to stress is shown relative to their expression level (in RPKM) under control conditions. **(C)** Distribution of genes down-regulated in response to stress is shown relative to their expression level (in RPKM) under stress conditions.
