# Supplementary material for: Transposable Elements Contribute to Activation of Maize Genes in Response to Abiotic Stress
Source: PLoS Genet. 2015 Jan 8;11(1):e1004915. doi: 10.1371/journal.pgen.1004915 (PMC4287451; doi:10.1371/journal.pgen.1004915)
Supplement: S2 Fig — Proportion of TE families differentially expressed in response to abiotic stress. Expression levels of 355 TE families were assessed and TE families were categorized as up-regulated (at least 2 fold increase in RPM values) or down-regulated (at least 2-fold decrease in RPM values) in response to abiotic stress. (PDF) [file pgen.1004915.s002.pdf]

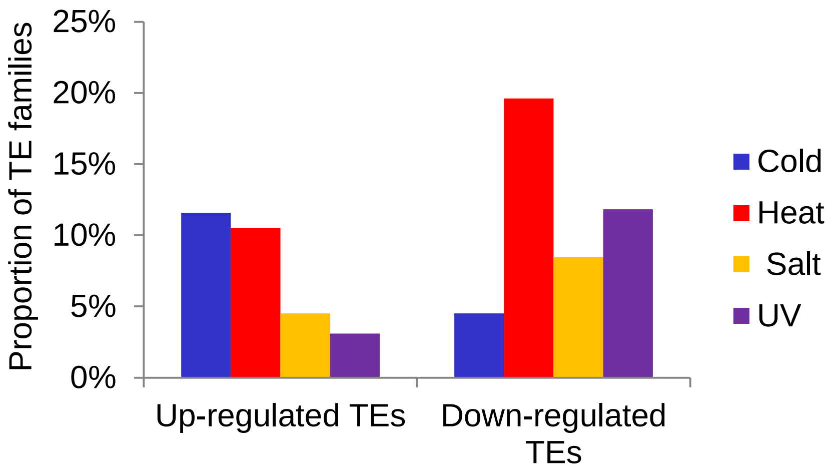

Figure S2. Proportion of TE families differentially expressed in response to abiotic stress. Expression levels of 355 TE families were assessed and TE families were categorized as up-regulated (at least 2 fold increase in RPM values) or down-regulated (at least 2-fold decrease in RPM values) in response to abiotic stress.
