# Supplementary material for: Transposable Elements Contribute to Activation of Maize Genes in Response to Abiotic Stress
Source: PLoS Genet. 2015 Jan 8;11(1):e1004915. doi: 10.1371/journal.pgen.1004915 (PMC4287451; doi:10.1371/journal.pgen.1004915)
Supplement: S3 Fig — Properties of TE insertions that condition stress-responsive expression. (A) In our initial screening we only analyzed TE insertions located within 1 kb of the TSS. Here we assessed the proportion of genes that exhibit stress-responsive expression for TE insertions located at different distances from the TSS (for the stress condition most associated with each TE family). Some of the TE families appear to only affect genes if they are inserted quite near the TSS while others can have influences at distances. (B) The CBF/DREB transcription factors have been associated with stress-responsive expression in a number of plant species [46]. We identified consensus CBF/DREB binding sites (A/GCCGACNT) in the consensus TE sequences (maizetedb.org) for the TEs associated with each of the stresses as well as in 40 randomly selected TEs that were not associated with gene expression responses to stress or 40 randomly selected 5 kb genomic regions. The proportion of sequences that contained a CBF/DREB binding site and the average number of sites per element are shown. The TEs associated with cold, heat and salt stress are all enriched for containing CBF/DREB binding sites. (PDF) [file pgen.1004915.s003.pdf]

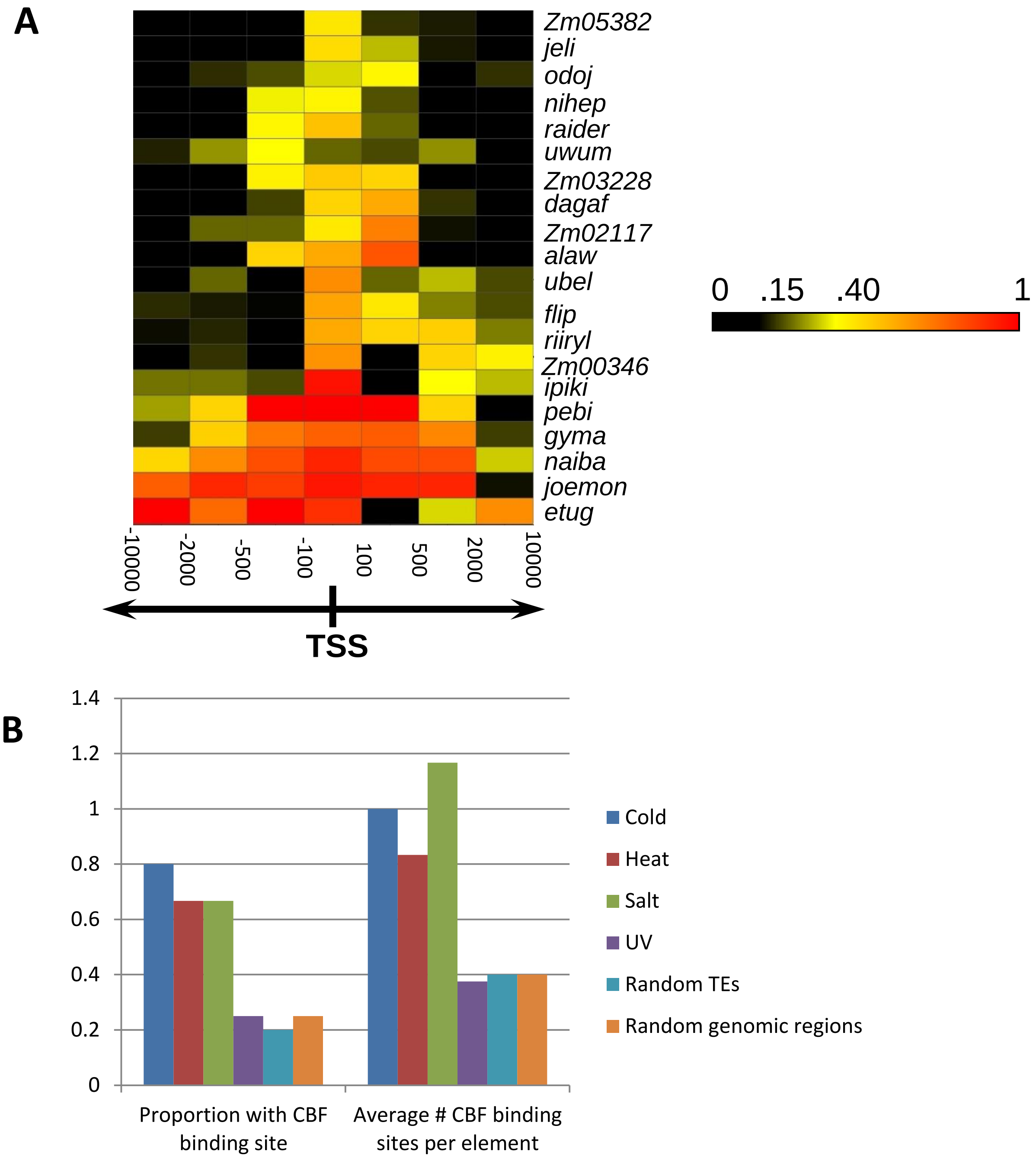

Figure S3. Properties of TE insertions that condition stress-responsive expression. (A) In our initial screening we only analyzed TE insertions located within 1kb of the TSS. Here we assessed the proportion of genes that exhibit stress-responsive expression for TE insertions located at different distances from the TSS (for the stress condition most associated with each TE family). Some of the TE families appear to only affect genes if they are inserted quite near the TSS while others can have influences at distances. (B) The CBF/DREB transcription factors have been associated with stress-responsive expression in a number of plant species [46]. We identified consensus CBF/DREB binding sites (A/GCCGACNT) in the consensus TE sequences (maizetedb.org) for the TEs associated with each of the stresses as well as in 40 randomly selected TEs that were not associated with gene expression responses to stress or 40 randomly selected 5kb genomic regions. The proportion of sequences that contained a CBF/DREB binding site and the average number of sites per element are shown. The TEs associated with cold, heat and salt stress are all enriched for containing CBF/DREB binding sites.
