# Supplementary material for: Transposable Elements Contribute to Activation of Maize Genes in Response to Abiotic Stress
Source: PLoS Genet. 2015 Jan 8;11(1):e1004915. doi: 10.1371/journal.pgen.1004915 (PMC4287451; doi:10.1371/journal.pgen.1004915)
Supplement: S4 Fig — TE insertions co-localized with TE-influenced stress-responsive genes frequently share the same part of the TE element. All naiba insestions near responsive (blue, n = 63) or non-responsive (red, n = 80) genes were aligned to the exemplar element. The proportion of elements that align to each portion of the sequence is plotted on the y-axis. The structure of the exemplar Naiba element structure is shown below the plot with various colors representing repeated regions of the element. The region that differentiates mostly between up-regulated and non-differentially expressed genes is a repeated region of the TE shown with a green arrow. (PDF) [file pgen.1004915.s004.pdf]

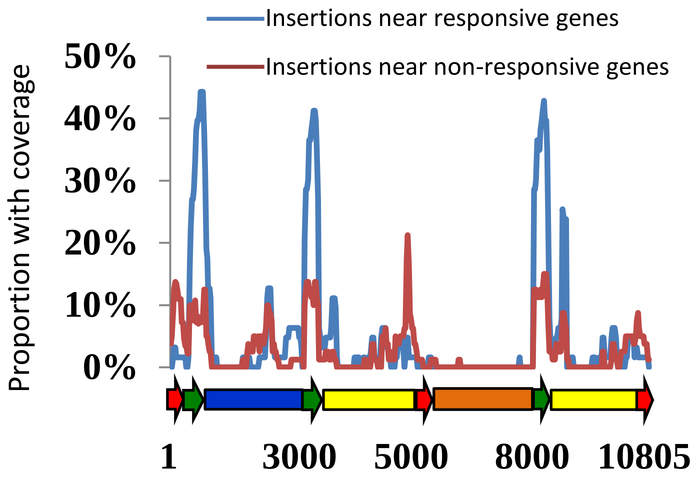

Figure S4. TE insertions co-localized with TE-influenced stress-responsive genes frequently share the same part of the TE element. All *naiba* insestions near responsive (blue, n=63) or non-responsive (red, n=80) genes were aligned to the exemplar element. The proportion of elements that align to each portion of the sequence is plotted on the y-axis. The structure of the exemplar *Naiba* element structure is shown below the plot with various colors representing repeated regions of the element. The region that differentiates mostly between up-regulated and non-differentially expressed genes is a repeated region of the TE shown with a green arrow.
