# Supplementary material for: Transposable Elements Contribute to Activation of Maize Genes in Response to Abiotic Stress
Source: PLoS Genet. 2015 Jan 8;11(1):e1004915. doi: 10.1371/journal.pgen.1004915 (PMC4287451; doi:10.1371/journal.pgen.1004915)
Supplement: S5 Fig — The conservation of stress-responsive expression of TE influenced genes varies for different families and different stresses. Proportion of genes up-regulated in B73 that are also up-regulated in Mo17 and Oh43 is shown for all four stresses for TE-influenced (black) and non-TE influenced (white) genes. (PDF) [file pgen.1004915.s005.pdf]

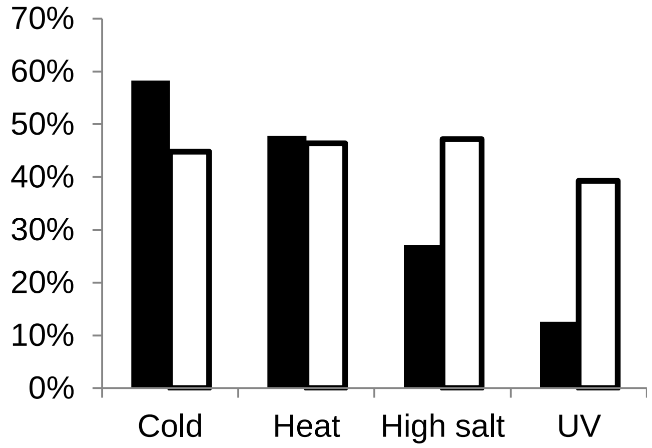


**Figure S5. The conservation of stress-responsive expression of TE influenced genes varies for different families and different stresses.** Percent of genes up-regulated in B73 that are also up-regulated in Mo17 and Oh43 is shown for all four stresses for TE-influenced (black) and non-TE influenced (white) genes.
